# Expert Comment Generation Considering Sports Skill Level Using a Large Multimodal Model with Video and Spatial-Temporal Motion Features

**DOI:** 10.3390/s25020447

**Published:** 2025-01-14

**Authors:** Tatsuki Seino, Naoki Saito, Takahiro Ogawa, Satoshi Asamizu, Miki Haseyama

**Affiliations:** 1 Graduate School of Information Science and Technology, Hokkaido University, N-14, W-9, Kita-ku, Sapporo 060-0814, Hokkaido, Japan; seino@lmd.ist.hokudai.ac.jp; 2 Office of Institutional Research, Hokkaido University, N-8, W-5, Kita-ku, Sapporo 060-0808, Hokkaido, Japan; saito@lmd.ist.hokudai.ac.jp; 3 Faculty of Information Science and Technology, Hokkaido University, N-14, W-9, Kita-ku, Sapporo 060-0814, Hokkaido, Japan; ogawa@lmd.ist.hokudai.ac.jp; 4 National Institute of Technology, Kushiro College, 2 Chome-32-1 Otanoshikenishi, Kushiro 084-0916, Hokkaido, Japan; asamizu@kushiro-ct.ac.jp

**Keywords:** expert comment generation, sports skill level, spatial-temporal attention graph convolutional network, large multimodal model

## Abstract

In sports training, personalized skill assessment and feedback are crucial for athletes to master complex movements and improve performance. However, existing research on skill transfer predominantly focuses on skill evaluation through video analysis, addressing only a single facet of the multifaceted process required for skill acquisition. Furthermore, in the limited studies that generate expert comments, the learner’s skill level is predetermined, and the spatial-temporal information of human movement is often overlooked. To address this issue, we propose a novel approach to generate skill-level-aware expert comments by leveraging a Large Multimodal Model (LMM) and spatial-temporal motion features. Our method employs a Spatial-Temporal Attention Graph Convolutional Network (STA-GCN) to extract motion features that encapsulate the spatial-temporal dynamics of human movement. The STA-GCN classifies skill levels based on these motion features. The classified skill levels, along with the extracted motion features (intermediate features from the STA-GCN) and the original sports video, are then fed into the LMM. This integration enables the generation of detailed, context-specific expert comments that offer actionable insights for performance improvement. Our contributions are twofold: (1) We incorporate skill level classification results as inputs to the LMM, ensuring that feedback is appropriately tailored to the learner’s skill level; and (2) We integrate motion features that capture spatial-temporal information into the LMM, enhancing its ability to generate feedback based on the learner’s specific actions. Experimental results demonstrate that the proposed method effectively generates expert comments, overcoming the limitations of existing methods and offering valuable guidance for athletes across various skill levels.

## 1. Introduction

Human motion skill assessment and feedback play a critical role in enhancing athletic performance through effective training and skill acquisition in sports. Achieving a high skill level often involves mastering complex movements, making it crucial to provide evaluations and feedback tailored to each individual’s unique characteristics. The sports training process hinges on two steps: identifying areas for improvement and implementing corrective actions [1]. Repetitive training under a coach’s guidance is significantly more effective than self-directed practice, as it allows for personalized feedback and adjustments based on the athlete’s performance [2]. However, personalized skill assessment and feedback require extensive specialized knowledge and experience, yet the number of coaches with such expertise is limited. Consequently, many learners lack access to skill-level-aware guidance.

One effective approach to providing more detailed and actionable advice is through free-form expert comments generated by a Large Language Model (LLM). This task of generating free-form expert comments is fundamentally different from general image and video captioning tasks. Unlike conventional captioning, which primarily describes observed activities [3,4,5,6,7,8,9,10], expert comment generation involves identifying key areas for improvement and suggesting actionable steps to enhance physical performance. The expert comment task requires a more profound understanding beyond conventional video captioning.

While further research is needed to address this challenge, ExpertAF [1] stands out as a notable exception. This method leverages an LLM to generate comprehensive, multifaceted feedback for learners. Specifically, it generates expert comments based on the video and assigned skill levels, retrieves relevant expert videos for comparison, and generates motion data after improvement. However, since ExpertAF primarily compares learners with predefined low and high skill levels, it may fall short in generating tailored comments for advanced players. Furthermore, when tokening motion information in ExpertAF, although it considers spatial information using a multilayer perceptron mixer, it does not fully account for temporal information. Considering spatial and temporal information, especially in sports, may improve learners’ recognition of action.

This paper proposes a novel expert comment generation method that incorporates sports skill levels. A Large Multimodal Model (LMM) integrated with video and spatial-temporal motion features is used to address the limitations of the previous method, i.e., ExpertAF. In the proposed method, motion features capturing the spatial-temporal dynamics of motion data are extracted and used for skill level classification. Several previous studies presented skill level estimation from videos in sports [11,12,13,14,15,16]. In addition, our earlier work introduced a Graph Convolutional Network (GCN)-based [17] skill level classification method [18]. Significantly, the Spatial-Temporal Attention GCN (STA-GCN) [19] effectively models human motion as a graph, where joints are represented as nodes and bones as edges, enabling it to capture spatial-temporal information. The attention mechanism in STA-GCN further enhances its performance by focusing on the most informative joints and time frames, thus improving the skill level classification accuracy. Therefore, the proposed method leverages STA-GCN to calculate motion features and classify skill levels. These outputs, along with the intermediate features from the STA-GCN and the original sports video, are used as inputs for expert comment generation. These inputs are tokenized and processed through the LMM, enabling the generation of detailed and natural language comments that go beyond simple numerical evaluations. For instance, instead of merely indicating a low score for a particular movement, the model can provide specific advice for the learners’ improvement, such as “Increase the range of motion in your right arm during the swing”. The key point of the proposed method is that it integrates the STA-GCN for skill level classification and the LMM for generating expert comments. The STA-GCN extracts motion features by capturing spatial and temporal information, enabling accurate classification. On the other hand, LMM combines these features with visual tokens and skill level data to generate actionable feedback tailored to the learner’s needs.

To summarize, the contributions of our method are described as follows:

- Utilization of skill level classification results as inputs to LMM
  Our method incorporates the skill level classification results from the STA-GCN into the LMM, ensuring that the generated expert comments are appropriately tailored to the learner’s skill level.
- Incorporation of motion features considering spatial-temporal information for LMM
  By integrating motion features that account for spatial-temporal information as inputs to the LMM, we enhance the model’s ability to generate detailed and context-specific feedback based on the players’ movements.

The proposed method improves upon existing approaches by leveraging motion features derived from human skeletal data that effectively capture spatial-temporal dynamics for skill level classification. It further utilizes these computed motion features and corresponding skill levels to generate insightful and personalized expert comments.

The proposed method integrates skill level classification and spatial-temporal motion features into an LMM, enabling the generation of expert comments tailored to individual performance. This approach addresses the limitations of existing methods by providing precise and actionable feedback.

This paper is organized as follows: Section 2 presents the proposed method in detail. Section 3 discusses the experimental results, offering a qualitative and quantitative evaluation of the approach. Finally, Section 4 presents the conclusions of this paper.

## 2. Skill-Level-Aware Expert Comment Generation

**Figure 1:**
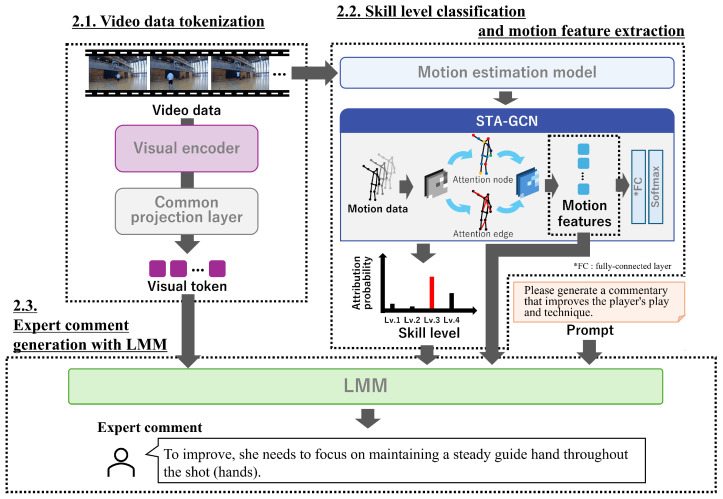
Overview of the proposed method for skill-level-aware expert comment generation. The input video is first processed through a visual encoder, which extracts and tokenizes video features via a shared projection layer. Simultaneously, motion data are derived from the sports video, and an STA-GCN computes skill level attribute probabilities and motion features. Leveraging these visual tokens, skill levels, and motion features, the proposed method generates expert comments through an LMM.

This section presents the proposed method for generating sports skill-level-aware expert comments using an LMM. An overview of the method is shown in Figure 1, and the procedure of the proposed method is shown in Algorithm 1. The proposed method introduces skill level classification into the expert comment generation process and generates feedback sentences. The proposed method contributions are (1) integrating skill level classification results and spatial-temporal motion features to enhance the contextual relevance of expert comments and (2) the design of a framework that enables the LMM to generate detailed, actionable feedback tailored to individual skill levels.

In this section, Section 2.1 describes the tokenization of sports video data. Section 2.2 details the classification of skill levels and the extraction of motion features. Finally, Section 2.3 presents the generation of expert comments using an LMM.
**Algorithm 1** Skill-Level-Aware Expert Comment Generation**Require:** Input video *V*, skeletal motion data *S*, pretrained STA-GCN, pretrained LMM **Ensure:** Generated expert comments *C* 1: **Visual Feature Extraction:** 2: Extract visual features $F_{v}\leftarrow \mathrm{VisualEncoder}\left(V\right)$ 3: Tokenize visual features $T_{v}\leftarrow \mathrm{Projection}\left(F_{v}\right)$ 4: **Motion Feature Extraction:** 5: Extract motion features $F_{m}\leftarrow \mathrm{STA}-\mathrm{GCN}\left(S\right)$ 6: Classify skill level $L\leftarrow \mathrm{SkillClassifier}\;(F_{m})$ 7: Tokenize motion features $T_{m}\leftarrow \mathrm{Projection}\left(F_{m}\right)$ 8: Tokenize skill level $T_{l}\leftarrow \mathrm{Projection}\left(L\right)$ 9: **Multimodal Integration:** 10: Combine tokens $T\leftarrow [T_{v},T_{m},T_{l}]$ 11: **Expert Comment Generation:** 12: Generate expert comments C←LMM(T,Prompt) 13: **return **
*C*

### 2.1. Video Data Tokenization

To process the input video *V*, we first tokenize it extracting visual features using a pre-trained visual encoder $f_{\mathrm{ve}}\left(\cdot \right)$. Our method employs the languagebind encoder [20] as visual encoder $f_{\mathrm{ve}}\left(\cdot \right)$, as it effectively maps different modalities into a unified textual feature space. This ensures that the visual representations are consistent and seamlessly compatible with textual processing. The extracted visual features are then transformed into token embeddings v through a common projection layer $f_{p}\left(\cdot \right)$. The tokenization process is formally defined as

(1) $$v=f_{p}\left(f_{\mathrm{ve}}\left(V\right)\right).$$

The proposed method effectively enables the visual features by converting the video *V* into token embeddings v.

### 2.2. Skill Level Classification and Motion Feature Extraction

A skeletal information estimation model was applied to the video to derive key point coordinates representing the human body’s joints and movements. The estimated skeletal information provides a structured and interpretable representation of human motion, which is crucial for accurately assessing skill levels in complex activities. As conventional methods for classifying complex motions, GCN-based approaches [17] have been proposed [21,22,23,24,25]. These methods effectively handle graph structures by modeling the relationships between human body joints, thereby enabling robust classification of intricate motions. In this study, we employ the STA-GCN [19], a model that has demonstrated effectiveness in skill level classification. By considering human joints as graph nodes $n_{t,i}\;(t=1,2,\ldots ,T;T$ being the number of frames for motion data, i=1,2,…,M;M being the number of human joints used to construct the graph), the graph is constructed by connecting nodes through interjoint and interframe edges. Interjoint edges are established on the basis of the adjacency relationships among human joints. In addition, interframe edges connect the same joints across consecutive frames, *t* and t+1, within the motion data. In STA-GCN, when the feature map of the *i*-th node at frame is $x\left(n_{t,i}\right)\in R^{D}$ (*D* being the dimensionality of the node features), the motion features $X_{\mathrm{in}}^{t}$ are defined as follows:

(2) $$X_{\mathrm{in}}^{t}={\left[x\left(n_{t,1}\right),\;x\left(n_{t,2}\right),\;\cdots ,\;x\left(n_{t,M}\right)\right]}^{⊤}\in R^{M\times D}.$$

**Figure 2:**
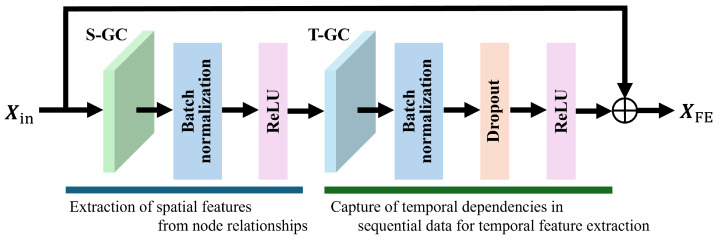
STGC block configuration. The STGC block is composed of five key network components; Spatial Graph Convolution (S-GC), which captures spatial relationships between joints; batch normalization, which normalizes feature distributions; ReLU, which introduces nonlinearity; Temporal Graph Convolution (T-GC), which captures temporal dependencies; and Dropout, which mitigates overfitting. Together, these components enable effective consideration of spatial-temporal features.

Next, $X_{\mathrm{in}}^{t}$ is input into the Spatial-Temporal Graph Convolutional (STGC) block, as shown in Figure 2, and a feature map $X_{\mathrm{FE}}\in R^{T\times M\times D}$ is computed. The STGC block was constructed using five key network components: Spatial Graph Convolution (S-GC), batch normalization [26], ReLU functions [27], Temporal Graph Convolution (T-GC), and Dropout [28]. As shown in the following, S-GC is designed to learn the spatial relationships between joints within the same frame.

(3) $$X_{\mathrm{out}}^{\mathrm{space}}=\sum_{h=1}^{H}W_{h}^{\mathrm{Edge}}\circ \left(\Lambda_{h}^{-1/2}\left(A_{h}^{\mathrm{space}}+I\right)\Lambda_{h}^{-1/2}\right)X_{\mathrm{in}}W_{h}^{\mathrm{Node}},$$

where $W_{h}^{\mathrm{Node}}$ and $W_{h}^{\mathrm{Edge}}$ (h=1,2,⋯,H;H being the number of adjacent nodes connected by intra-body edges) denote the weight matrices of the nodes and edges, respectively, and $A_{h}^{\mathrm{space}}$ denotes the adjacency matrix in the spatial direction. The symbol “∘” denotes the Hadamard product, and $I\in R^{M\times M}$ denotes the identity matrix. Furthermore, $\Lambda_{h}\in R^{M\times M}$ denotes a diagonal matrix whose diagonal elements are $\Lambda_{ii}=\sum_{\rho }^{M}\left(A_{i}+I_{i\rho }\right)$. It takes as input a node feature matrix representing joint features and an adjacency matrix that defines the graph structure. The adjacency matrix is normalized, and self-loops are added to enhance feature propagation. Additionally, a learnable edge importance mask is applied to weigh the significance of each connection dynamically. The convolution operation is then performed using the normalized adjacency matrix and node features, enabling the model to extract critical spatial features between joints. The output feature matrix captures spatial dependencies and serves as input for subsequent processing stages. On the other hand, the T-GC is shown in the following.

(4) $$x^{\mathrm{time}}\left(n_{t,i}\right)=\sum_{\tau =-\lfloor \Gamma /2\rfloor }^{\left\lfloor \Gamma /2\right\rfloor }\alpha_{\tau }\circ x\left(n_{t-\tau ,i}\right)\in R^{D},$$

where Γ denotes the size of the T-GC kernel and $\alpha_{\tau }\in R^{D}$ denotes the weight vector of the T-GC. The T-GC focuses on modeling temporal relationships between the same joints across consecutive frames. It takes as input a feature tensor representing spatial relationships across frames and applies a one-dimensional convolution along the temporal axis. The convolution uses a kernel size Γ to capture dynamic changes and motion patterns over time. This process generates a feature tensor that emphasizes temporal characteristics, allowing the model to effectively learn the time-dependent aspects of actions.

The STGC blocks in the proposed method build upon previously reported techniques [18,19]. These STGC blocks were selected for their demonstrated effectiveness in modeling spatial and temporal dependencies in human motion data. Specifically, the STGC block integrates S-GC to capture relationships between joints within a frame and T-GC to model motion dynamics across consecutive frames. The advantage of using the STGC block is its ability to effectively capture the intricate interplay of spatial structures and temporal motion. In summary, the STGC block provides a well-validated framework for spatial-temporal feature extraction. Within this block, a new feature map $X_{\mathrm{FE}}$ that retains spatial-temporal information is calculated through the combined operations of S-GC and T-GC.

Using the feature map $X_{\mathrm{FE}}$, the proposed method extracts attention nodes that signify the importance of each joint for skill level classification and attention edges that capture the relationships between key joints during motion. The attention node $J\left(X_{\mathrm{FE}}\right)\in R^{1\times M\times M}$ is computed by applying multiple 1×1 convolution layers, batch normalization, upsampling, and a sigmoid activation function. Furthermore, the attention edge $E\left(X_{\mathrm{FE}}\right)\in R^{M\times M}$, which encodes only the critical connections relevant to classification, is derived by applying multiple 1×1 convolution layers and Global Average Pooling (GAP) [29] to the feature map $X_{\mathrm{FE}}$. Batch normalization, a tanh function, and the ReLU function are then used to suppress irrelevant elements by converting nonessential values to zero. Using the attention edge $E(X_{\mathrm{FE}})$ and the attention node $J(X_{\mathrm{FE}})$, the method computes the enhanced feature map $X_{AN}$, which emphasizes the joints important for skill level classification, as follows:

(5) $$X_{\mathrm{AN}}=J\left(X_{\mathrm{FE}}\right)X_{\mathrm{FE}}.$$

$X_{\mathrm{AN}}$ emphasizes critical spatial relationships by weighting joints and edges based on their importance for skill level classification. The feature map $X_{\mathrm{AM}}$ is calculated by convolving the attention edges with $X_{\mathrm{AN}}$ as shown in the following equation:

(6) $$X_{\mathrm{AM}}=\sum_{\theta =1}^{\Theta }\left(\Lambda_{\theta }^{-1/2}\left(A_{\theta }^{\mathrm{AN}}+I\right)\Lambda_{\theta }^{-1/2}\right)X_{\mathrm{AN}}W_{\theta }^{\mathrm{AN}},$$

where $A_{\theta }^{\mathrm{AN}}\in R^{M\times M}(\theta =1,2,\ldots ,\Theta$; Θ being the number of attention edges) represents the adjacency matrix, and $W_{\theta }^{\mathrm{AN}}\in R^{M\times M}$ is the weight matrix. In addition, $I\in R^{M\times M}$ denotes the identity matrix, and $\Lambda_{\theta }\in R^{M\times M}$ is a diagonal matrix. Next, the spatial feature map $X_{\omega }$ is computed by convolving the feature maps $X_{\mathrm{FE}}$ and $X_{\mathrm{AN}}$. The sum of $X_{\mathrm{AM}}$ and $X_{\omega }$ is calculated as the final feature map $X_{\mathrm{out}}=X_{\mathrm{AM}}+X_{\omega }$. This formulation represents the combination of two complementary feature maps. $X_{\mathrm{AM}}$ captures the refined spatial-temporal information derived through the attention-enhanced adjacency matrix, highlighting key motion dynamics. $X_{\omega }$ represents direct spatial feature information, ensuring that critical spatial details are preserved. The summation of these two components integrates the strengths of both perspectives, yielding a comprehensive representation of the motion data. This approach reflects the need to balance global spatial structures with localized, attention-driven refinements for accurate classification. The motion features $X_{\mathrm{Motion}}\in R^{D_{\mathrm{Motion}}}$ ($D_{\mathrm{Motion}}$ (being the output dimension of motion features) are then obtained using GAP and a fully connected layer. In the proposed method, we input the motion features $X_{\mathrm{Motion}}$ into the LMM. Furthermore, by applying GAP, a fully connected layer, and a softmax function to the motion features, the probabilities for each skill level are computed, enabling skill level classification.

### 2.3. Expert Comment Generation with LMM

The core component of our method is the LMM, which generates expert comments by inputting visual tokens v, motion features $X_{\mathrm{Motion}}$, and skill level information as inputs. The LMM is specifically designed to process multiple input modalities, enabling it to generate detailed and context-specific feedback. The inputs to the LMM are summarized as follows:

- Tokenized Video Features: Encoded representations of the visual content, capturing relevant aspects of the performance.
- Motion Features: Detailed motion information extracted from the perception branch of the STA-GCN, highlighting fundamental movements and joints.
- Skill Levels: Outputs from the attention branch of the STA-GCN, providing insights into the learner’s skill level.
- Prompts: Predefined text inputs or instructions are designed to guide expert comments.

The LMM integrates these inputs to produce free-form expert comments that are specifically tailored to the learner’s skill level and unique performance characteristics. The integration of information into the LMM involves tokenizing visual features, motion features, and skill level classification results into embeddings compatible with the LMM’s input format. Visual features are extracted using a pre-trained visual encoder, motion features are derived from STA-GCN, and skill level classifications are tokenized to explicitly represent the player’s proficiency. These tokenized features are concatenated into a unified multimodal token sequence, which is paired with a predefined prompt to guide the LMM. This process ensures the LMM can utilize the integrated data to generate contextually relevant and actionable feedback tailored to the player’s performance. Next, to ensure contextual relevance and maintain consistency, the LMM leverages predefined prompts to guide the language generation process. The prompt used in the proposed method is

*Please generate a commentary that improves the player’s play and technique based on the video, skill level, and motion features below. In parentheses (), specify the body part that needs improvement.*

The LMM effectively synthesizes multimodal inputs to deliver detailed and actionable expert comments, including tokenized video features, motion features, skill levels, and predefined prompts.

### 2.4. Training Procedure

In the proposed method, the training process focuses exclusively on optimizing the parameters of the projection layer $f_{p}\left(\cdot \right)$ while keeping the STA-GCN and LMM fixed. Specifically, both the STA-GCN and the LMM are pre-trained models with parameters that remain unchanged during training. The purpose of training the projection layer $f_{p}\left(\cdot \right)$ is to align the visual features with the textual space of the LMM, enabling the generation of accurate and context-specific expert comments. The primary objective is to minimize the discrepancy between the generated expert comments and the ground truth annotations. This alignment is achieved by optimizing the projection layer $f_{p}\left(\cdot \right)$ to produce token embeddings that, when processed by the pre-trained LMM, yield expert comments that closely match the ground truth annotations. The training process employs the sequence generation loss $L_{\mathrm{token}}$, which calculates the negative log-likelihood of the generated expert comment sequence based on the following inputs:

(7) $$L_{\mathrm{token}}=-\log p\left(Y_{A}|v,Y_{\mathrm{GT}}\right),$$

where $Y_{A}$ is the expert comment sequence generated by the LMM, and $Y_{\mathrm{GT}}$ denotes the textual tokens obtained from ground truth expert comments, tokenized using the GPT-4o tokenizer. This tokenization process converts the ground truth comments into a sequence of textual tokens in the same format as the output in LMM.

Since the STA-GCN and LMM are pre-trained with fixed parameters, the loss function $L_{\mathrm{token}}$ is minimized with respect to the parameters of the projection layer $f_{p}\left(\cdot \right)$. Our training procedure focuses on optimizing the projection layer to reduce the sequence generation loss $L_{\mathrm{token}}$, ensuring that the generated expert comments are precise and tailored to the learner’s performance. By leveraging the pre-trained capabilities of the STA-GCN and LMM, the training process effectively aligns the visual features with the language model. This approach enables efficient training and facilitates the generation of high-quality expert comments informed by visual and motion data.

## 3. Experimental Results

In this section, we evaluate the effectiveness of the proposed method for generating expert comments from sports videos. The experiments were conducted using basketball videos to generate expert feedback. In addition, the generation performance was quantitatively assessed, complemented by a qualitative evaluation of the generated expert comments to illustrate the method’s effectiveness. In this section, Section 3.1 describes the experimental settings. Section 3.2 details the quantitatively evaluated generated expert comment performance. Finally, Section 3.3 presents the qualitative evaluation results.

### 3.1. Experimental Settings

**Figure 3:**
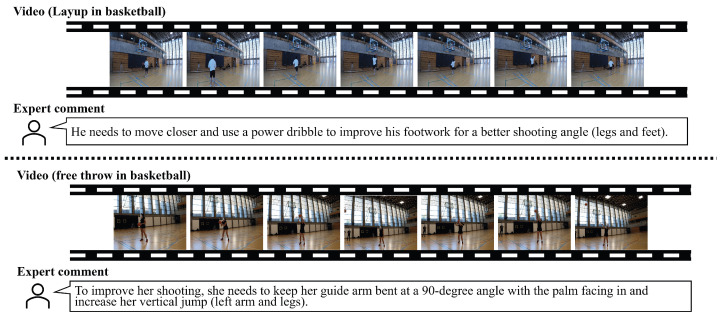
Examples of a basketball video and the expert comments included in the Ego-Exo4D.

This experiment utilized basketball videos from the Ego-Exo4D [30] dataset, sports videos with sufficient skill levels, and expert comments. The motion data were extracted from these videos using MediaPipe (https://developers.google.com/mediapipe, accessed on 15 November 2024). MediaPipe is an open-source framework designed for extracting motion data, enabling tasks such as pose estimation, hand-tracking, and gesture recognition. Examples of a basketball video and its corresponding expert comments are illustrated in Figure 3. For our experiments, we extracted 100 frames before and after each timestamp of expert comments, treating each segment as an individual sample. In addition, the expert comments in the dataset do not specify which body parts are involved (e.g., legs, arms), which is crucial for providing detailed feedback. To address this limitation, we performed a preprocessing step to clarify which body parts the expert commentary pertains to, simplifying the expert comments. We used GPT-4 omni (GPT-4o) (https://openai.com/index/hello-gpt-4o/, accessed on 15 November 2024) for the LMM of summarizing expert comments and proposed methods.

In this experiment, STA-GCN and LMM are pre-trained models. The STA-GCN is pre-trained on a dataset of motion data to classify skill levels. Specifically, we used the 154 basketball motion data and four skill levels included in the Ego-Exo4D dataset to pre-train the model. For the LMM, we utilized GPT-4o, a pre-trained large language model optimized for multimodal tasks.

We used the basketball data from the Ego-Exo4D dataset in our experiments, which comprised 1313 samples. In this experiment, we randomly divided the 1313 basketball video samples in the Ego-Exo4D dataset into about 90% (1213 samples) for training data and about 10% (100 samples) for test data.

The following three evaluation indices were used in the experiment to evaluate the expert comment generation performance.

- BLEU-4 [31] (↑)
  Measures the *n*-gram overlap between the generated comments and the ground truth, focusing on 4-gram matches. BLEU-4 is defined as follows:
  (8) $$\mathrm{BLEU}-4=\mathrm{BP}\times \exp\left(\frac{1}{4}\sum_{k=1}^{4}\log z_{k}\right),$$
  where $z_{k}$ represents the precision of the *n*-grams, indicating the proportion of the *n*-gram matches between the generated sentence and the ground truth. BP is the brevity penalty, which applies a penalty when the generated sentence is shorter than the ground truth. It is defined as follows:
  (9) $$\mathrm{BP}=\left\{\begin{array}{cc} \exp\left(1-\frac{r}{c}\right) & \mathrm{if}\;c<r,\\ 1 & \mathrm{otherwise}, \end{array}\right.$$
  where *c* represents the length of the generated sentence determined by its word count, with *r* representing the length of the ground truth sentence calculated similarly.
- METEOR [32] (↑)
  Considers the precision and recall of unigrams, incorporating synonym matches and stemming. The METEOR score is defined as follows:
  (10) $$\mathrm{METEOR}=F_{\mathrm{mean}}\times \left(1-\mathrm{Penalty}\right),$$
  where $F_{\mathrm{mean}}$ is the harmonic mean of the precision and recall,
  (11) $$F_{\mathrm{mean}}=\frac{10\times \mathrm{Recall}\times \mathrm{Precision}}{\mathrm{Recall}+9\times \mathrm{Precision}},$$
  (12) $$\mathrm{Recall}=\frac{\mathrm{Number}\;\mathrm{of}\;\mathrm{matched}\;\mathrm{words}}{\mathrm{Number}\;\mathrm{of}\;\mathrm{words}\;\mathrm{in}\;\mathrm{the}\;\mathrm{generated}\;\mathrm{expert}\;\mathrm{comment}},$$
  (13) $$\mathrm{Precision}=\frac{\mathrm{Number}\;\mathrm{of}\;\mathrm{matched}\;\mathrm{words}}{\mathrm{Number}\;\mathrm{of}\;\mathrm{words}\;\mathrm{in}\;\mathrm{the}\;\mathrm{ground}\;\mathrm{truth}\;\mathrm{expert}\;\mathrm{comments}}.$$
  The weights for the harmonic mean are based on the conventional literature [32]. In addition, Penalty represents the fragmentation penalty, which penalizes disordered matches. It is computed as
  (14) $$\mathrm{Penalty}=\gamma \times \left(\frac{\mathrm{chunks}}{\mathrm{matches}}\right).$$
  where the chunks denotes the number of contiguous sequences of matching words, matches is the total number of matched words, and γ is a parameter.
- ROUGE-L [33] (↑)
  The longest common subsequence between the generated comments and the reference is evaluated, capturing the sentence level structure.
  (15) $$\mathrm{ROUGE}-L=\frac{\left(1+\beta^{2}\right)\times {\mathrm{LCS}}_{\mathrm{Precision}}\times {\mathrm{LCS}}_{\mathrm{Recall}}}{{\mathrm{LCS}}_{\mathrm{Precision}}+\beta^{2}\times {\mathrm{LCS}}_{\mathrm{Recall}}}.$$
  The parameter β controls the trade-off between precision and recall. ${\mathrm{LCS}}_{\mathrm{Precision}}$ is defined as the length of the Longest Common Subsequence (LCS) divided by the length of the generated sequence, whereas ${\mathrm{LCS}}_{\mathrm{Recall}}$ is the length of the LCS divided by the length of the ground truth. They are computed as
  (16) $${\mathrm{LCS}}_{\mathrm{Precision}}=\frac{\mathrm{LCS}}{\mathrm{length}\;\mathrm{of}\;\mathrm{generated}\;\mathrm{sequence}},$$
  (17) $${\mathrm{LCS}}_{\mathrm{Recall}}=\frac{\mathrm{LCS}}{\mathrm{length}\;\mathrm{of}\;\mathrm{ground}\;\mathrm{truth}}.$$

Higher values in these metrics indicate better performance and closer alignment with the ground truth expert comments.

To evaluate the performance of the proposed method (PM), this experiment uses seven comparative methods: PM without incorporating skill level information from the STA-GCN (CM1), PM without motion feature extraction from the STA-GCN (CM2), PM using only visual token embeddings without skill level information and motion features (CM3), PM using only motion features and skill level information without visual token embeddings from the video (CM4), PM without both skill level information and visual token embeddings, using only motion features (CM5), Video-LLaVA [5] (CM6), and ExpertAF [1] (CM7). Ablation studies were conducted to assess the contributions of different components in PM. Table 1 provides the correspondence for each component of the CMs1-5. These experiments help evaluate the individual and combined effects of skill level data, motion features, and visual tokenization on expert comment generation performance. Video-LLaVA is a representative video captioning method that generates descriptive captions of video content. It serves as a baseline for comparing general video captioning performance in expert comment generation. ExpertAF, conversely, is an existing expert comment generation method designed primarily for novice learners and does not consider the learner’s skill level. Comparing the PM with ExpertAF allows us to assess the importance of skill level awareness in providing more nuanced feedback. By comparing the PM with these variants and existing approaches, we aim to demonstrate the effectiveness of each component in the PM (skill level, motion features, and visual tokenization).

### 3.2. Quantitative Evaluation of Generated Expert Comments

Table 2 presents the BLEU-4, METEOR, and ROUGE-L scores for basketball expert comment generations from the Ego-Exo4D dataset. The PM achieved the highest scores across all evaluation metrics, demonstrating its effectiveness in generating high-quality expert comments. This superior performance of the PM can be attributed to several key components integrated into the model. First, a comparison between PM and CM1 shows that incorporating skill level information significantly enhances performance. This skill level allows the model to tailor comments that are more nuanced and appropriate to the learner’s expertise, enabling it to account for the varying complexities of different plays and resulting in more contextually relevant feedback. Second, comparisons between PM, CM2, and CM3 highlight the effectiveness of incorporating motion data that consider spatial-temporal information. Motion features capture the dynamic aspects of the scenes, such as player movements and interactions, which are crucial for generating insightful expert comments. These motion features, with their spatial-temporal understanding, allow the model to describe not only static visuals but also the flow of actions within the scenes. Third, the evaluation of PM against CM4 and CM5 underscores the importance of visual information. Visual tokenization enables the model to effectively encode spatial features from the visual data. This visual representation allows the model to generate comments that accurately reflect the visual content of the basketball scenes, leading to improved performance.

Furthermore, PM outperforms CM6, a general video captioning method, indicating that PM is better suited for expert comment generation. This superior performance is likely due to PM’s specialized architecture, which integrates domain-specific features, such as skill level, visual tokenization, and motion features elements, not considered by general video captioning models. Finally, comparing the PM with CM7, a prior expert comment generation method, confirms that PM achieves superior performance. ExpertAF is currently the only existing method specifically designed for generating expert comments in the context of sports skill level evaluation. Therefore, we included a direct comparison with ExpertAF in Table 2 to evaluate the advantages of our method. Other methods in the broader fields of video captioning or motion analysis, while advanced, are not directly applicable to this task due to their lack of consideration for skill-level-specific feedback or their inability to process the integration of spatial-temporal motion features with skill level classification. For instance, methods like Video-LLaVA generate descriptive captions for videos but do not provide actionable, context-aware feedback tailored to an athlete’s performance. By comparing the proposed method with ExpertAF, we demonstrated its superiority in generating detailed, actionable, and skill-level-aware comments. PM combines skill level classification, visual encoding, and motion understanding, generating more informative and tailored expert comments than existing methods. In summary, the enhanced performance of our PM results from the synergistic integration of skill level information, visual tokenization, and spatial-temporal motion features. These components collectively enable the generation of valid and contextually expert comments during the learning process by employing skill level and motion features considering spatial-temporal information. This study successfully meets the research objectives by improving expert comment generation through a model that accounts for both the skill level and motion features, considering spatial-temporal information.

### 3.3. Qualitatively Evaluation Results

**Figure 4:**
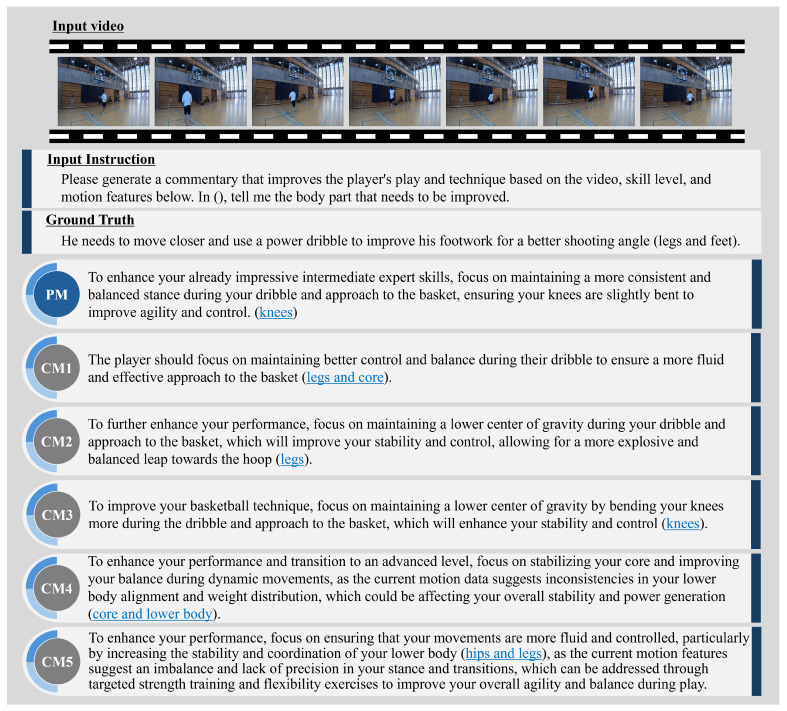
Comparison of expert comment sentences generated by the PM and CM1-5. The parts highlighted in blue indicate the important human body parts or their surrounding human body parts in the ground truth sentence.

**Figure 5:**
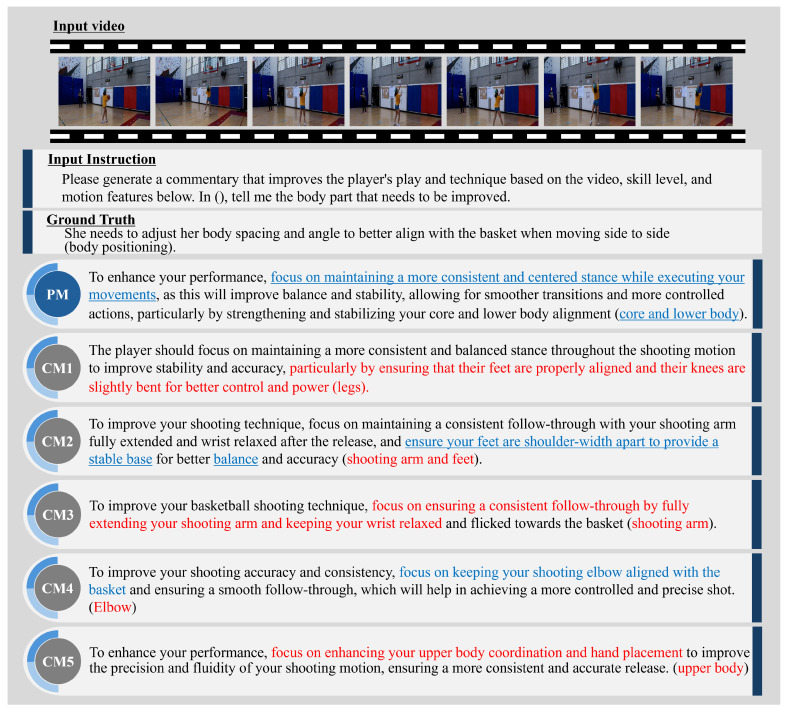
PM and CM1-5 expert comments on a video of a basketball layup shot when CMs that do not include skill level do not produce valid expert comments. The sentences highlighted in blue indicate actions that require improvement and share similar meanings with those in the ground truth sentence. In contrast, the sections highlighted in red in CM1-5 also indicate actions needing improvement but have different meanings compared to the ground truth sentence.

**Figure 6:**
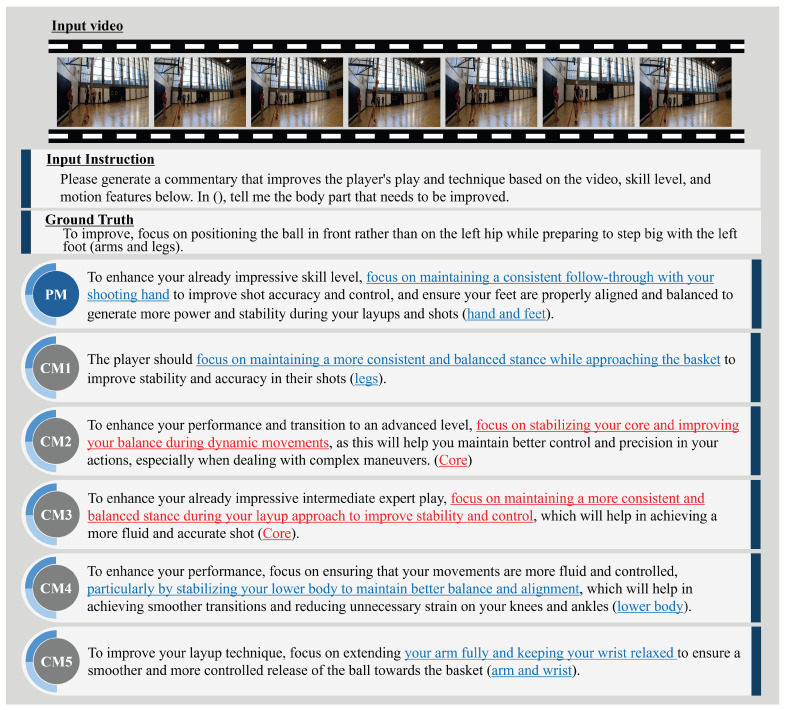
PM and CM1-5 expert comments on a video of a basketball layup shot when CMs that do not include motion do not produce valid expert comments. The sentences highlighted in blue represent actions requiring improvement and closely align in meaning with the corresponding ground truth sentence. In contrast, the sections highlighted in red in CM2 and CM3 also identify actions needing improvement but differ in meaning compared to the ground truth sentence.

**Figure 7:**
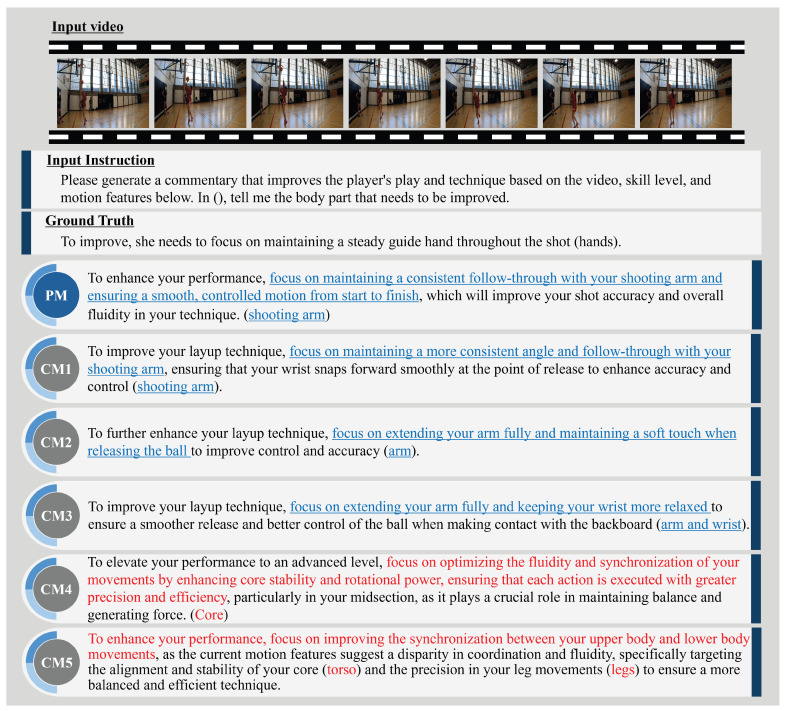
PM and CM1-5 expert comments on a video of a basketball layup shot when CMs that do not include video do not produce valid expert comments. The sentences highlighted in blue represent actions requiring improvement and closely align in meaning with the corresponding ground truth sentence. In contrast, the sections highlighted in red in CM4 and CM5 also identify actions needing improvement but differ in meaning compared to the ground truth sentence.

This subsection presents examples of expert comment results and evaluates the PM’s effectiveness. Figure 4, Figure 5, Figure 6 and Figure 7 illustrate expert comments generated by the PM and through ablation studies (CM1-5). The results in Figure 4 indicate that both the PM and the comparative methods generate free-form comments with semantic content equivalent to the ground truth expert comments. In Figure 5, methods incorporating skill levels (PM, CM2, and CM4) produce comments that are semantically aligned with the ground truth expert feedback. In contrast, methods that do not consider skill levels (CM1, CM3, and CM5) generate comments referencing unrelated body parts, deviating from the expected feedback. These findings confirm that incorporating skill level information significantly enhances the relevance of expert comment generation. Furthermore, Figure 6 demonstrates that methods utilizing video information (PM, CM1, CM4, and CM5) identify areas for improvement closely aligned with those specified in the expert comments. In contrast, the CM without video information (CM2 and CM3) outputs are different from the outputs of actual important human body parts. This underscores the importance of video data in generating accurate and context-specific expert comments. Finally, Figure 7 shows that methods incorporating motion information (PM, CM1, CM2, and CM3) effectively identify areas for improvement that closely correspond to the expert comments. In contrast, methods without motion information (CM4 and CM5) generate feedback that focuses on less relevant body parts, deviating from the expected outcomes. This suggests that motion information can be crucial in expert comment generation. These findings suggest that skill level, video, and motion information are significant factors in expert comment generation. Furthermore, PM effectively identified basketball-specific movements and provided comments to assist players in skill improvement.

**Figure 8:**
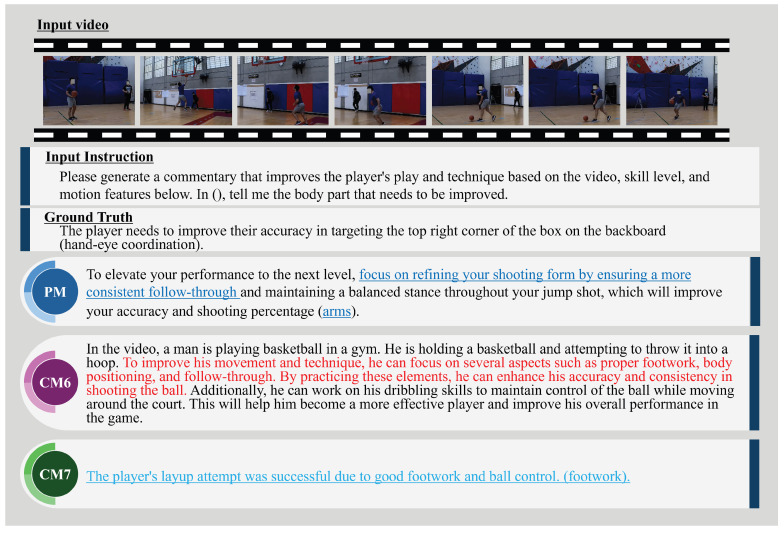
Examples of expert comments generated by the PM, CM6, and CM7 from a video of a layup shot in basketball. The PM (highlighted in blue) provides comments focused on the arms, closely matching the intended meaning of the ground truth. CM6 (in red) delivers generic advice with irrelevant details, such as dribbling, reflecting a lack of focus on the specific action. CM7 (in light blue) provides relevant yet superficial comments, recognizing good footwork but overlooking opportunities to address hand-eye coordination and targeting accuracy for further improvement.

**Figure 9:**
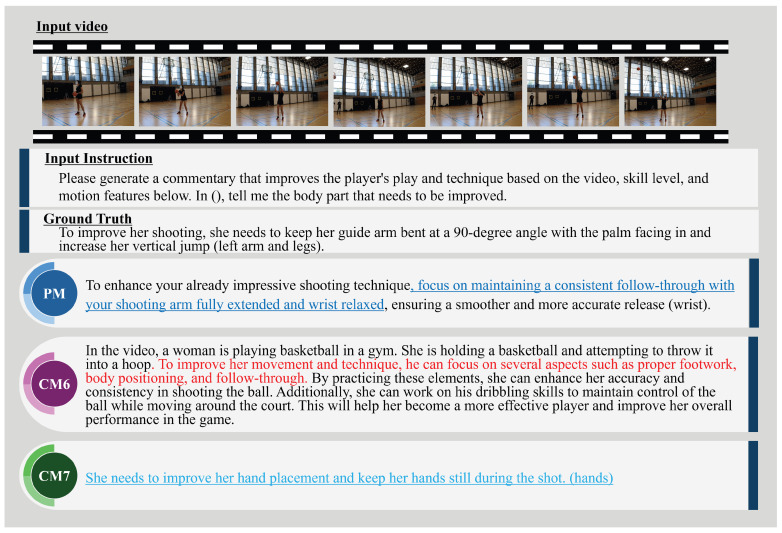
Examples of expert comments generated by the PM, CM6, and CM7 from a video of a free throw in basketball. The PM (highlighted in blue) delivers precise and actionable feedback, emphasizing consistent follow-through and relaxed wrist positioning, closely aligning with the meaning of the ground truth sentence. In contrast, CM6 (highlighted in red) provides a broad and generic scene description, often including irrelevant details. At the same time, CM7 (highlighted in light blue) offers relevant but superficial advice, lacking depth and specificity in addressing shooting techniques.

In addition, Figure 8 and Figure 9 show examples of expert comments generated by the PM, CM6 (Video-Llava), and CM7 (ExpertAF). To further highlight the advantages of the proposed method, we show the qualitative results in Figure 8 and Figure 9 by color-coding key parts of the generated comments. This visual enhancement distinguishes specific, actionable feedback provided by the proposed method, which aligns closely with the ground truth. In contrast, comments from comparative methods were often generic or lacked actionable details, making the differences in performance more apparent. Specifically, actionable feedback provided by the proposed method, such as “maintain a consistent follow-through” or “adjust your arm positioning”, is highlighted in blue. In contrast, generic or less actionable comments from comparative methods are represented in red and light blue. The results in Figure 8 show that the PM provides specific and actionable advice, directly addressing the player’s shooting technique. The PM emphasizes refining the shooting form by ensuring a consistent follow-through and maintaining a balanced stance during the jump shot, with the goal of improving accuracy and shooting percentage. This feedback focuses on the arms, aligning with the key aspects necessary for enhancing shooting performance. While the PM does not explicitly mention every detail in the ground truth, it offers complementary advice that contributes to better accuracy, demonstrating a nuanced understanding of the fundamental skills required for improvement. In contrast, Video-Llava provides a general description of the scene, offering broad advice on various aspects, such as footwork, body positioning, follow-through, and even dribbling skills. However, its feedback lacks specificity and includes irrelevant details that are not pertinent to the depicted action, revealing a limitation in generating focused, context-specific expert comments. ExpertAF correctly identified that the player performed a layup and acknowledged the success due to good footwork and ball control, with a primary focus on footwork. While the comment is relevant and positive, it lacks constructive feedback for further improvement. It does not address areas where the player could enhance their layup technique, such as utilizing the backboard or improving hand-eye coordination. The results in Figure 9 show that the PM provides specific and actionable advice to improve the shooting technique. It highlights the importance of a consistent follow-through with a fully extended arm and a relaxed wrist, both critical aspects of an effective basketball shot. This feedback closely aligns with the ground truth, emphasizing arm positioning and shot mechanics, while also mentioning details like keeping the guide arm bent and increasing the vertical jump. Video-Llava is the same as Figure 9 and provides a broad scene description and generic advice, including irrelevant elements such as dribbling skills, indicating a lack of focus and specificity. In contrast, ExpertAF offers more general suggestions about hand placement and keeping hands still during the shot. While relevant, this feedback lacks depth and does not delve into the specific mechanics that could significantly enhance the player’s performance.

These qualitative evaluation results demonstrate that the PM outperforms in generating expert comments closely aligned with the ground truth. Specifically, in Figure 8, the proposed method provides detailed advice, such as emphasizing consistent follow-through and maintaining a balanced stance, directly addressing areas for performance improvement. In contrast, CM7’s comments, while relevant, are more general and lack actionable suggestions, such as specific techniques or adjustments for improvement. Similarly, in Figure 9, the proposed method highlights critical aspects of shooting mechanics, including arm positioning and relaxed wrist movements, which align closely with the ground truth and offer clear guidance for players. However, CM7 does not fully capture the nuances required for skill refinement. These qualitative differences underscore the contribution of STA-GCN in capturing spatial-temporal information and integrating skill level information, which allows the proposed method to generate feedback that is not only contextually relevant but also more effective in guiding players toward specific performance improvements. By effectively incorporating skill level, visual tokenization, and motion features, the PM provides targeted feedback that aids players in improving their skills. This underscores the importance of these factors in expert comment generation and highlights the PM’s effectiveness in sports training applications.

## 4. Conclusions

In this paper, we proposed a method for generating skill-level-aware expert comments using an LMM incorporating spatial-temporal motion features. The PM effectively addresses the limitations of conventional expert comment generation methods, which often struggle to provide meaningful feedback for advanced learners. Additionally, recognizing the critical role of spatial-temporal motion information in sports, we incorporated motion features that capture these dynamics. Specifically, skill levels and spatial-temporal motion features were computed from video using STA-GCN, and the LMM generated expert comments by leveraging video data, skill levels, and motion features. Our method enables the generation of expert comments tailored to a player’s skill level. Additionally, extracting spatial-temporal motion features from the intermediate layers of the STA-GCN enhances the quality of the generated expert comments.

## Figures and Tables

**Table 1:** The correspondence table for each component of the proposed and comparative methods.

	Skill Level	Motion	Video
PM	✓	✓	✓
CM1		✓	✓
CM2	✓		✓
CM3			✓
CM4	✓	✓	
CM5		✓	

**Table 2:** Quantitative evaluation of expert comment generation using the proposed and comparative methods with basketball video in the Ego-Exo4D dataset. Bold text indicates the highest performance for each evaluation metric.

	BLEU-4	METEOR	ROUGE-L
PM	**1.75 × 10^−2^**	**0.256**	**0.156**
CM1	$1.57\times 10^{-2}$	0.245	0.138
CM2	$1.46\times 10^{-2}$	0.256	0.147
CM3	$1.56\times 10^{-2}$	0.253	0.153
CM4	$1.12\times 10^{-2}$	0.195	0.112
CM5	$1.07\times 10^{-2}$	0.211	0.120
CM6	$9.50\times 10^{-3}$	0.145	0.118
CM7	$1.66\times 10^{-2}$	0.256	0.153

## Data Availability

Publicly available datasets were analyzed in this study. The public datasets used in our experiment are available at https://ego-exo4d-data.org/ (accessed on 19 November 2024).
